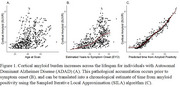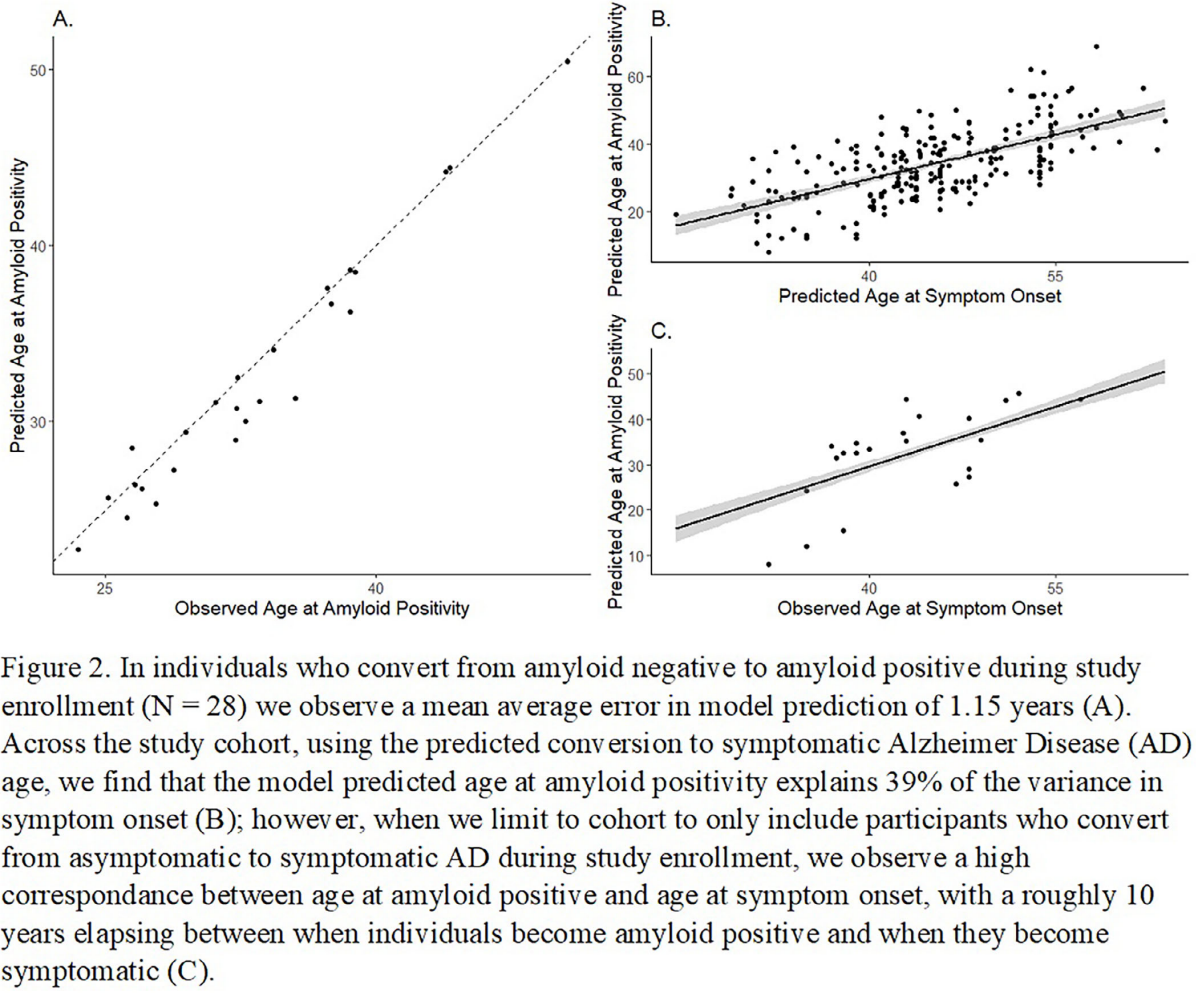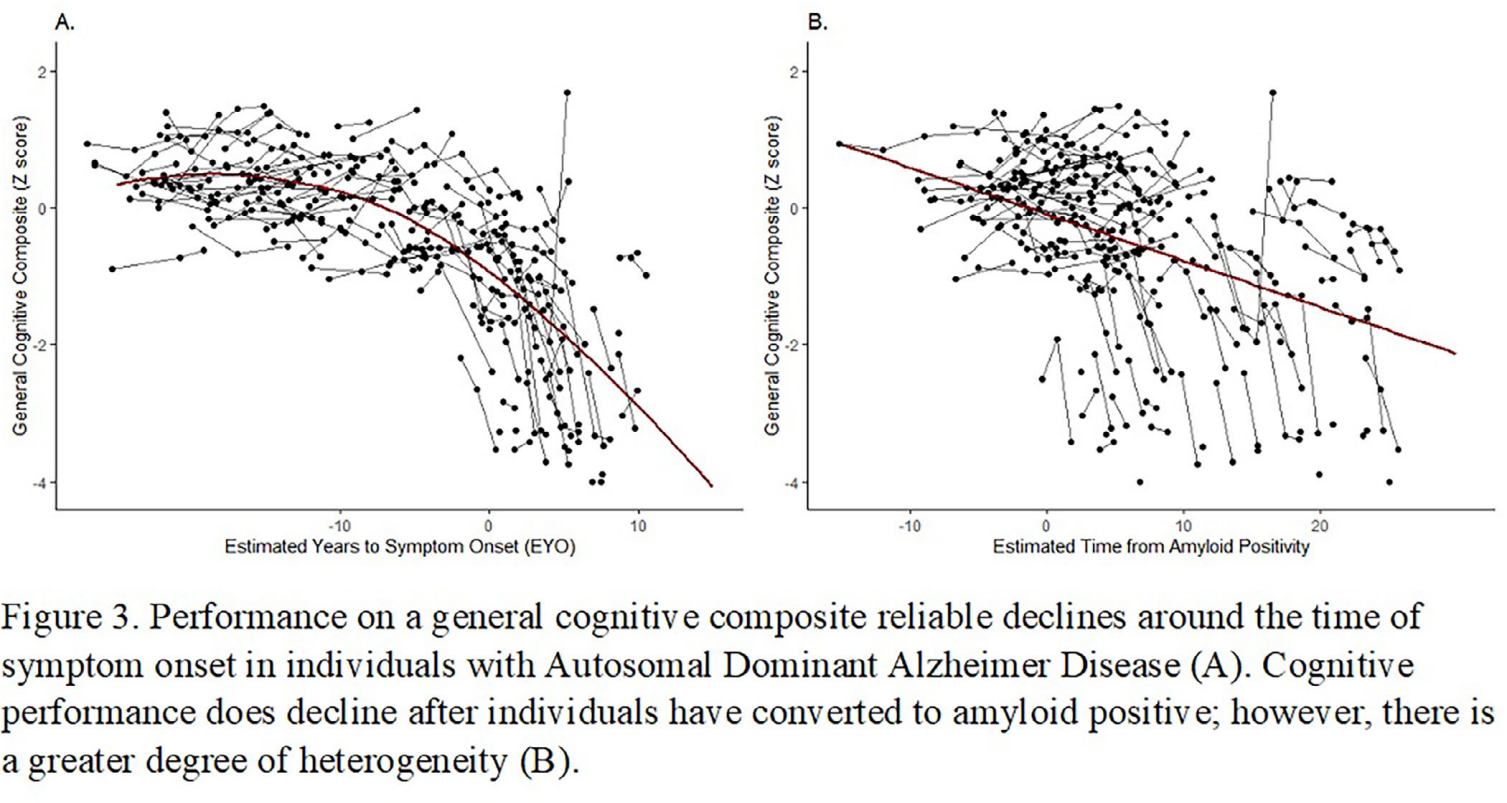# Validation of Amyloid Chronicity in Autosomal Dominant Alzheimer Disease

**DOI:** 10.1002/alz70856_103008

**Published:** 2025-12-25

**Authors:** Julie K. Wisch, Nicole S. McKay, Matthew D Zammit, Bradley T Christian, Stephanie A. Schultz, Peter R Millar, Nicolas R. Barthélemy, Natalie S Ryan, Alan E. Renton, Lisa Vermunt, Nelly Joseph‐Mathurin, Zahra Shirzadi, Jeremy F. Strain, Patricio Chrem, Alisha Daniels, Jasmeer P. Chhatwal, Carlos Cruchaga, Laura Ibanez, Mathias Jucker, Gregory S Day, Jae‐Hong Lee, Johannes Levin, Jorge J. Llibre‐Guerra, David Aguillon, Jee Hoon Roh, Charlene Supnet‐Bell, Chengjie Xiong, Suzanne E. Schindler, Guoqiao Wang, Yan Li, Robert Koeppe, Clifford R. Jack, John C. Morris, Eric McDade, Randall J. Bateman, Tammie L.S. Benzinger, Beau Ances, Tobey J. Betthauser, Brian A. Gordon, the Dominantly Inherited Alzheimer Network

**Affiliations:** ^1^ Washington University in St. Louis School of Medicine, St. Louis, MO, USA; ^2^ Washington University in St. Louis, School of Medicine, St. Louis, MO, USA; ^3^ Waisman Center, University of Wisconsin‐Madison, Madison, WI, USA; ^4^ Massachusetts General Hospital, Brigham and Women's Hospital, Harvard Medical School, Boston, MA, USA; ^5^ Department of Neurology, Washington University School of Medicine, St. Louis, MO, USA; ^6^ Department of Neurology, Washington University in St. Louis School of Medicine, St. Louis, MO, USA; ^7^ UK Dementia Research Institute at UCL, London, United Kingdom; ^8^ Icahn School of Medicine at Mount Sinai, New York, NY, USA; ^9^ Alzheimer Center Amsterdam, Neurology, Vrije Universiteit Amsterdam, Amsterdam UMC location VUmc, Amsterdam, North Holland, Netherlands; ^10^ Washington University School of Medicine, St. Louis, MO, USA; ^11^ Massachusetts General Hospital, Harvard Medical School, Boston, MA, USA; ^12^ Fleni, Buenos Aires, Argentina; ^13^ Massachusetts General Hospital, Boston, MA, USA; ^14^ Hope Center for Neurological Disorders, Washington University in St. Louis, St. Louis, MO, USA; ^15^ Hope Center for Neurological Disorders, St. Louis, MO, USA; ^16^ German Center for Neurodegenerative Diseases (DZNE), Tuebingen, Germany; ^17^ Mayo Clinic in Florida, Jacksonville, FL, USA; ^18^ Department of Neurology, Asan Medical Center, University of Ulsan College of Medicine, Seoul, Korea, Republic of (South); ^19^ Department of Neurology, LMU University Hospital, LMU Munich, Munich, Munich, Germany; ^20^ Neurosciences Group of Antioquia, University of Antioquia, Medellín, Colombia; ^21^ Washington University School of Medicine in St. Louis, St. Louis, MO, USA; ^22^ Washington University in St. Louis, St. Louis, MO, USA; ^23^ Washington University School of Medicine, St Louis, MO, USA; ^24^ University of Michigan, Ann Arbor, MI, USA; ^25^ Mayo Clinic, Rochester, MN, USA; ^26^ Knight Alzheimer Disease Research Center, Washington University School of Medicine, St. Louis, MO, USA; ^27^ Washington University St. Louis School of Medicine, St. Louis, MO, USA; ^28^ The Tracy Family SILQ Center, St. Louis, MO, USA; ^29^ University of Wisconsin‐Madison School of Medicine and Public Health, Madison, WI, USA; ^30^ Washington University School of Medicine, Saint Louis, MO, USA

## Abstract

**Background:**

Alzheimer Disease (AD) pathology evolves over decades, and understanding this progression is critical to the understanding of the disease and timing therapeutic interventions. Since individuals with Autosomal Dominant AD (ADAD) develop symptoms around the same age as their parent, it is possible to predict symptom onset and stage individuals by their estimated years to symptom onset (EYO). This approach does not generalize to other forms of AD, thus there is a pressing need for the timecourse of ADAD to be defined in broadly relevant terms.

The objective of this project is to validate the Sampled Iterative Local Approximation (SILA) algorithm in a cohort with a known disease timecourse. SILA generates an estimate of time from amyloid positivity (A_time_) based on longitudinal PET data.

**Method:**

We evaluated A_time_ in a longitudinal ADAD sample (*N* = 316) with PET PiB data in three ways. First, we compared predicted age at amyloid positive (A+) to observed age at A+ for individuals who became A+ during enrollment. Next, using linear regression, we compared estimated age at A+ to estimated age at symptom onset (EYO=0). Finally, we used generalized additive models to compare the amount of variance in concurrent cognitive performance explained both A_time_ and EYO.

**Result:**

We observed a mean average error of 1.15 years between actual age at A+ (*N* = 26) and the SILA‐predicted A_time_. Across all participants, SILA‐estimated age at A+ explained 39% of the variance in estimated age at symptom onset (*β* = 0.918, *p* < 0.0001). Finally, we observed a nonlinear association between cognition and both A_time_ and EYO. A_time_ explained 19% of the variance in the general cognitive composite while EYO explained 43% of the variance.

**Conclusion:**

SILA produces a valid estimate of time‐from‐amyloid positivity in ADAD. This work allows for disease stage in ADAD to be compared to staging for broad forms of AD, which was not previously possible using EYO. However, this work also illustrates that there is a high degree of heterogeneity in preclinical disease duration that is not explained by amyloid alone.